# Supplementary material for: Strong coupling of collective optical resonances in dielectric metasurfaces
Source: Light Sci Appl. 2025 Nov 24;14:387. doi: 10.1038/s41377-025-02076-6 (PMC12641030; doi:10.1038/s41377-025-02076-6)
Supplement: Supplementary file 1 — Supporting Information: Strong coupling of collective optical resonances in dielectric metasurfaces [file 41377_2025_2076_MOESM1_ESM.pdf]

Supporting Information: Strong coupling of collective optical resonances in dielectric metasurfaces

Izzatjon Allayarov[1,2,3], Vittorio Aita[4], Diane J. Roth[4,5], Boaz van Casteren[4], Anton Yu. Bykov[4,6], Andrey B. Evlyukhin[3,7*], Anatoly V. Zayats[4*], Antonio Calà Lesina[1,2,3*]

[1*]Hannover Centre for Optical Technologies, Leibniz University Hannover, Nienburger Str. 17, Hannover, 30167, State, Germany.
[2]Institute of Transport and Automation Technology, Leibniz University Hannover, An der Universität 2, Garbsen, 30823, Germany.
[3]Cluster of Excellence PhoenixD, Leibniz University Hannover, Welfengarten 1A, Hannover, 30167, Germany.
[4]Department of Physics and London Centre for Nanotechnology, King's College London, Strand, London, WC2R 2LS, UK.
[5]QinetiQ, Cody Technology Park, Ively Road, Farnborough, GU14 0LS, UK.
[6]NanoPhotonics Centre, Cavendish Laboratory, Department of Physics, University of Cambridge, , Cambridge, CB3 0HE, UK.
[7]Institute of Quantum Optics, Leibniz University Hannover, Welfengarten 1, Hannover, 30167, Germany.

*Corresponding author(s). E-mail(s): evlyukhin@iqo.uni-hannover.de; anatoly.zayats@kcl.ac.uk; antonio.calalesina@hot.uni-hannover.de;

1 Lattice sums

The explicit forms of the lattice sums (S_x, S_y, S_z) and the coupling parameter g_x can be written as [1]

$$g_x = -k_{\text{sur}} \sum_{n=0}^{\infty} \sum_{m=0}^{\infty} x_n F_{nm} \left(\frac{\mathrm{i}k_{\text{sur}}}{r_{nm}} - \frac{1}{r_{nm}^2} \right) \neq 0 \quad \text{if} \quad \sin\theta \neq 0, \tag{S1}$$

$$S_x = k_{\text{sur}}^2 \sum_{n=0}^{\infty} \sum_{m=0}^{\infty} F_{nm} \left(1 + \frac{\mathrm{i}}{k_{\text{sur}} r_{nm}} - \frac{1}{k_{\text{sur}}^2 r_{nm}^2} - \frac{x_n^2}{r_{nm}^2} - \frac{3\mathrm{i}x_n^2}{k_{\text{sur}} r_{nm}^3} + \frac{3x_n^2}{k_{\text{sur}}^2 r_{nm}^4} \right), \tag{S2}$$

$$S_y = k_{\text{sur}}^2 \sum_{n=0}^{\infty} \sum_{m=0}^{\infty} F_{nm} \left(1 + \frac{\mathrm{i}}{k_{\text{sur}} r_{nm}} - \frac{1}{k_{\text{sur}}^2 r_{nm}^2} - \frac{y_m^2}{r_{nm}^2} - \frac{3\mathrm{i}y_m^2}{k_{\text{sur}} r_{nm}^3} + \frac{3y_m^2}{k_{\text{sur}}^2 r_{nm}^4} \right), \tag{S3}$$

$$S_z = k_{\text{sur}}^2 \sum_{n=0}^{\infty} \sum_{m=0}^{\infty} F_{nm} \left(1 + \frac{\mathrm{i}}{k_{\text{sur}} r_{nm}} - \frac{1}{k_{\text{sur}}^2 r_{nm}^2} \right), \tag{S4}$$

where k_{sur} is the wave number in a medium with a refractive index of n_{sur}, $r_{nm} = |\mathbf{r}_{nm}| = \sqrt{x_n^2 + y_m^2}$, $x_n = Pn$, $y_m = Pm$ and $F_{nm} = \exp[\mathrm{i}k_{\text{sur}}(r_{nm} + x_n \sin\theta)]/(4\pi r_{nm})$, n and m are integer numbers, P is the period of a square lattice, θ is the polar angle of the of incidence. Note that the $n = m = 0$ term must be excluded from the above sums. For the sake of convenience, we assume that all particles are placed on the xy-plane at $z = 0$.

2 Supplementary figures

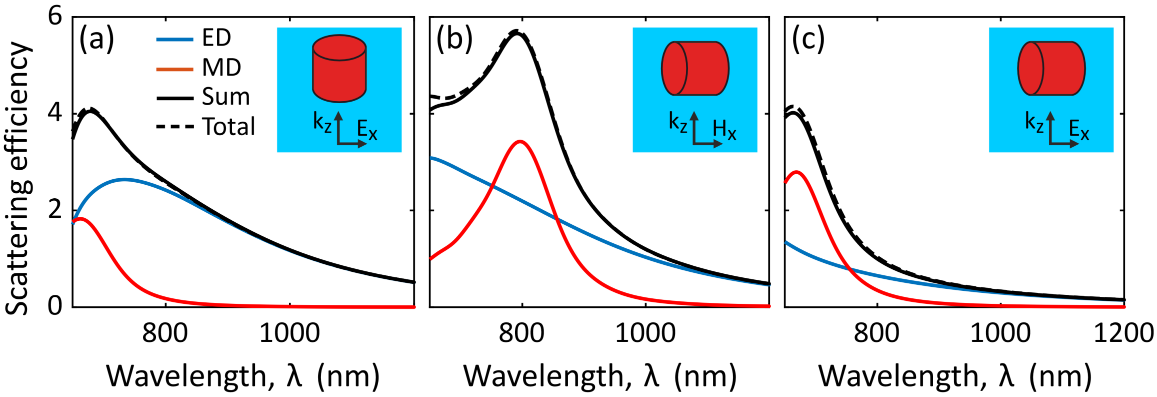

Figure S1 Simulated scattering efficiency of a single polycrystalline Si nanodisk for different excitation conditions indicated in the insets. The individual disk has a diameter $D = 220$ nm, height $H = 100$ nm, and a refractive index of the surrounding medium n_{sur}=1.45. ED (blue): electric dipole contribution, MD (red): magnetic dipole contribution, Sum: ED + MD, Total: all multipoles. Comparison of Sum and Total shows that only the dipolar response of the nanodisk is important in the considered spectral range.

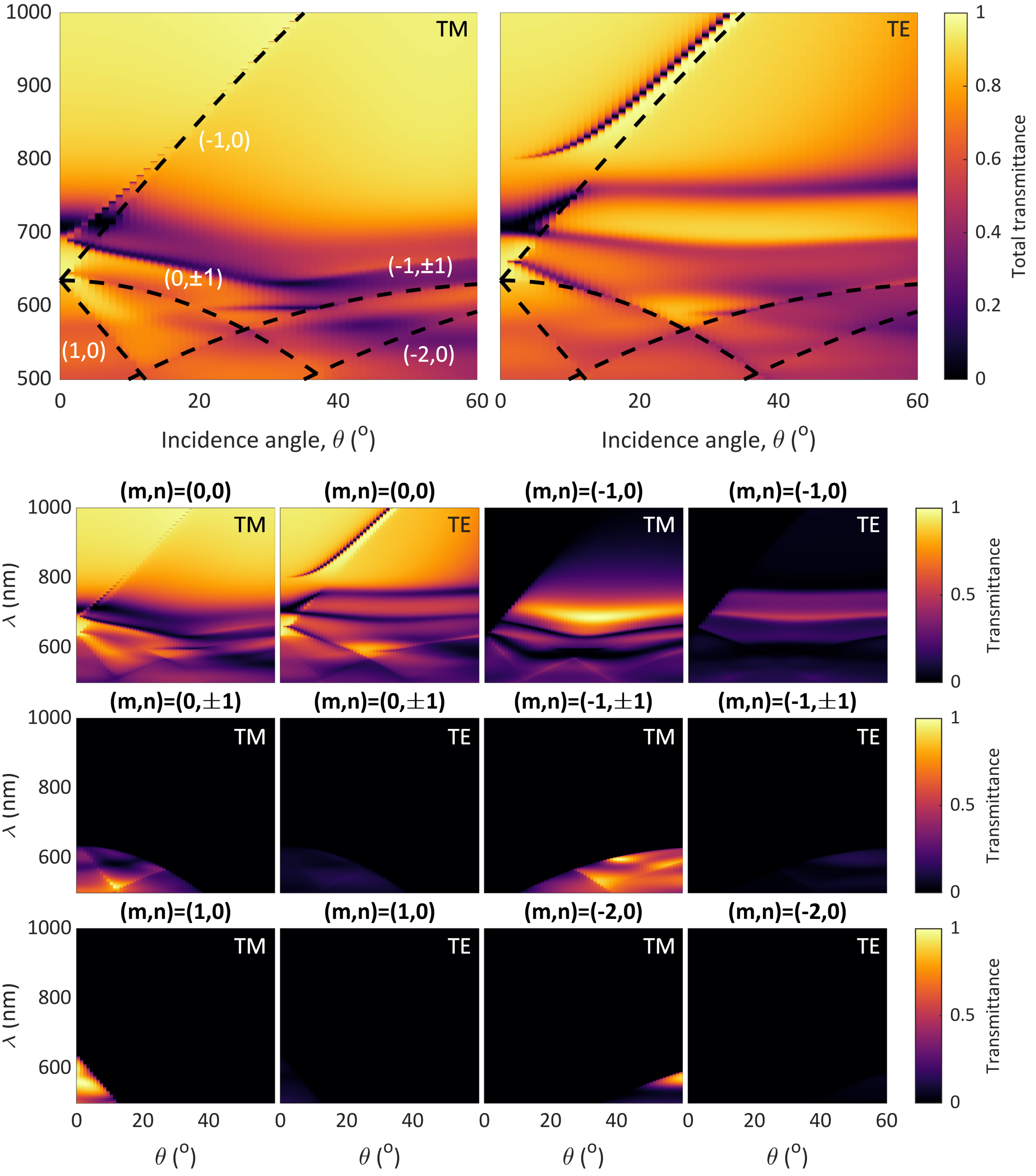

Figure S2 Total transmittance (top row panels) of the metasurface considered in Fig. 2 of the main text for TM- and TE-polarised illumination. The black dashed lines indicate the positions of all Rayleigh anomaly orders (orders are given in brackets and the same for both TM and TE cases) for the considered incidence angle and wavelength range. The panels below present the decomposition of the total transmittance into the grating order contributions.

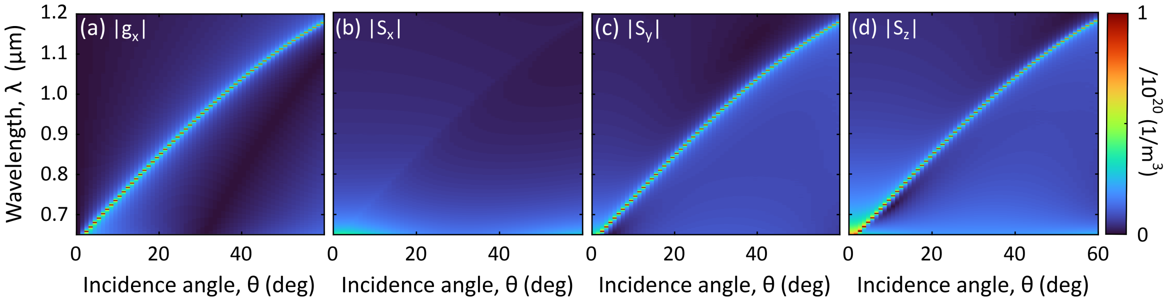

Figure S3 Simulated dispersions with respect to polar angle of incidence θ of the absolute value of (a) the coupling parameter g_x and the dipole lattice sums (b) S_x, (c) S_y, and (d) S_z. The period of a square lattice is $P = 440$ nm and the refractive index of the environment is n_{sur}=1.45.

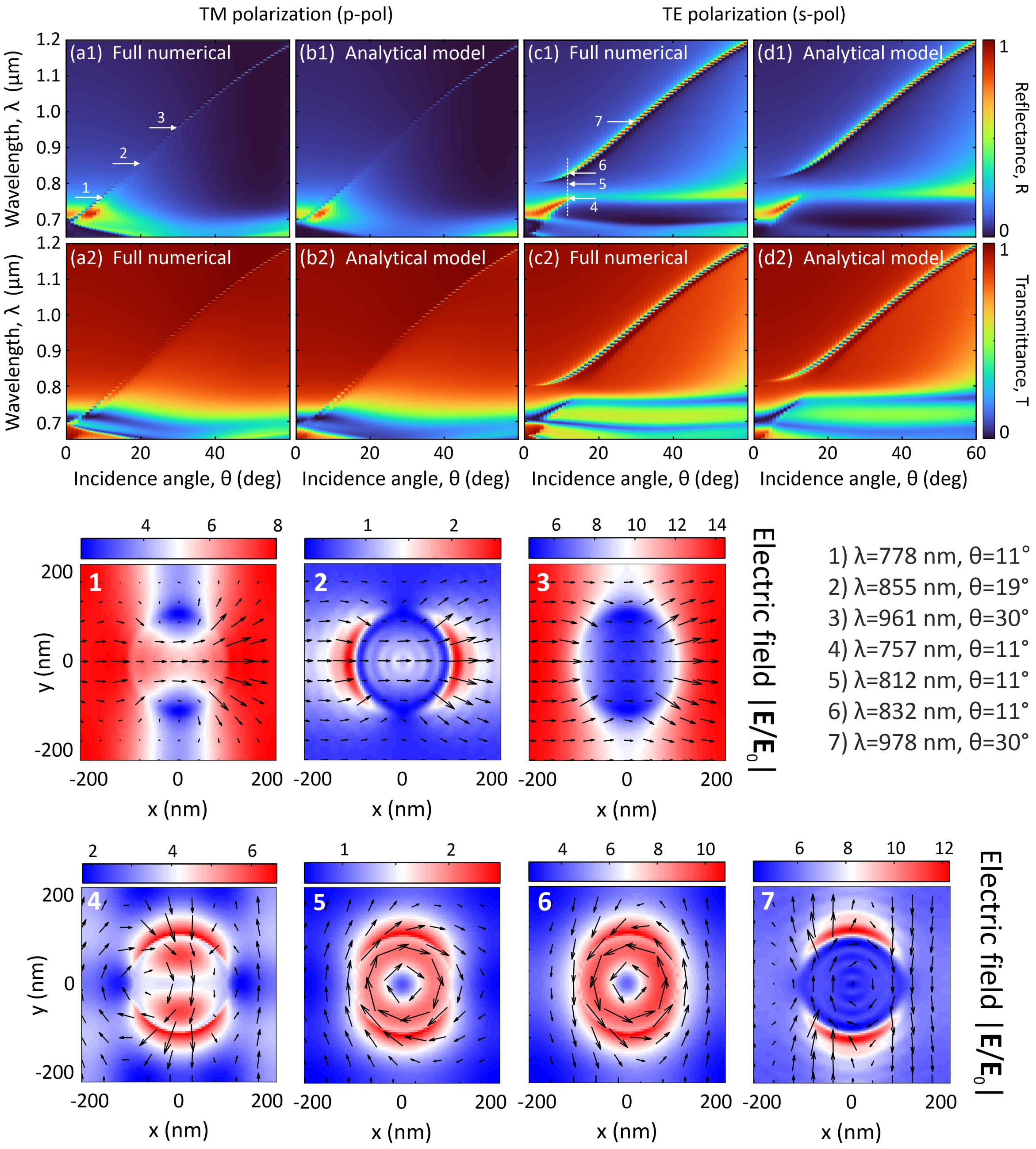

Figure S4 Comparison of the theoretical results from the full numerical and the coupled-dipole model simulations for the reflectance and transmittance dispersion of the metasurface immersed in a homogeneous environment ($n_{\mathrm{sur}} = n_{\mathrm{sub}} = n_{\mathrm{sup}}$=1.45) for (a,b) TM- and (c,d) TE-polarised illumination. Only the main diffraction orders are shown. The bottom panels show the normalized electric field distributions at the positions indicated by numbers in panels (a1) and (c1). E_0 is the electric field of the incident wave. The white dashed circle indicates a Si nanodisk. The metasurface, with a period of the square lattice P = 440 nm, consists of nanodisks with a diameter D = 220 nm and a height H = 100 nm. Surrounding refractive index is n_{sur}=1.45.

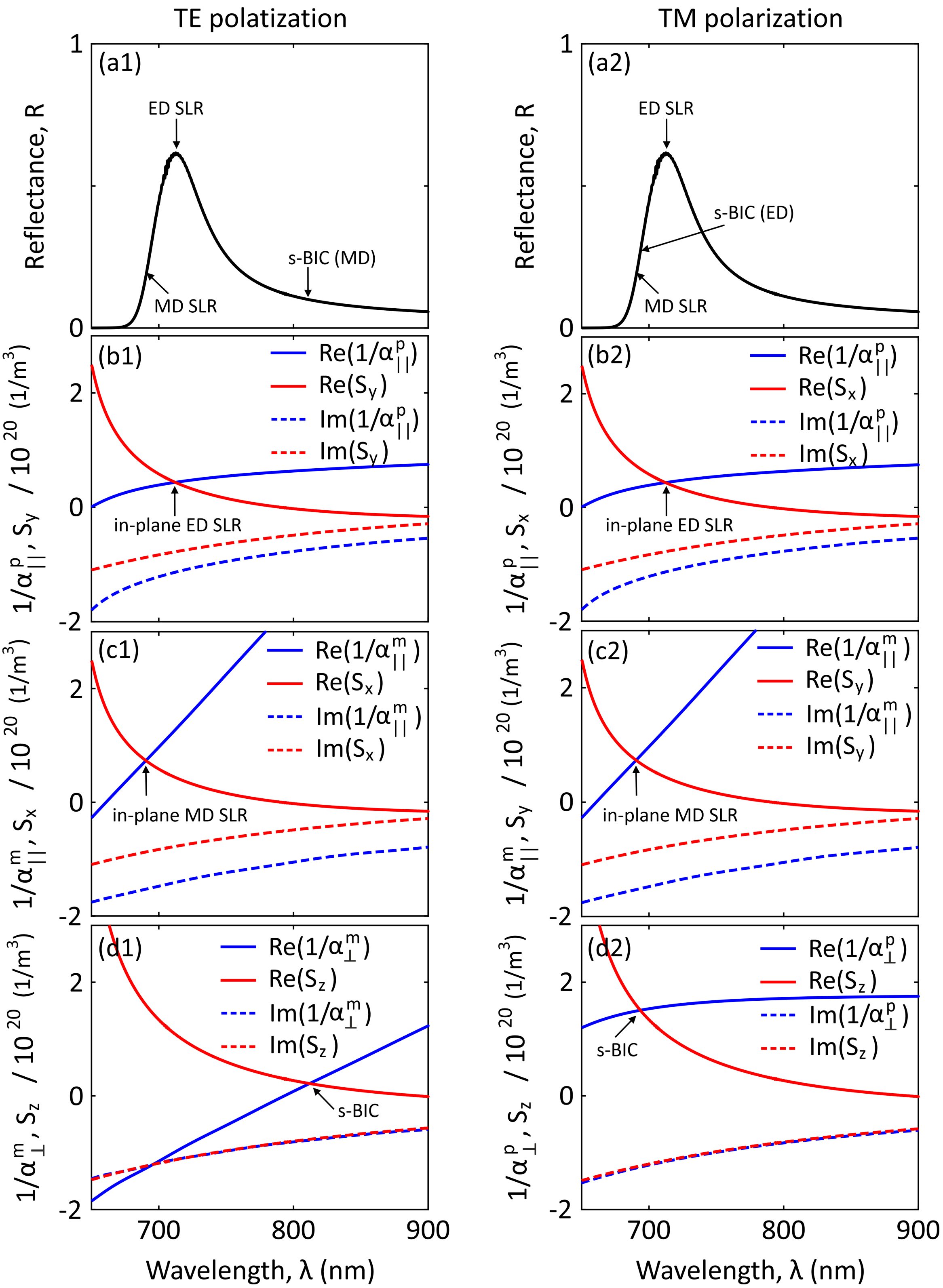

Figure S5 (a) Simulated total reflectance with the indicated positions of the SLR and the sBIC for (1) TE- and (2) TM-polarised illumination at normal incidence (θ=0). (b–d) The conditions leading to the spectral features associated with (b) in-plane ED SLR, (c) in-plane MD SLR, and (d) symmetry-protected BIC.

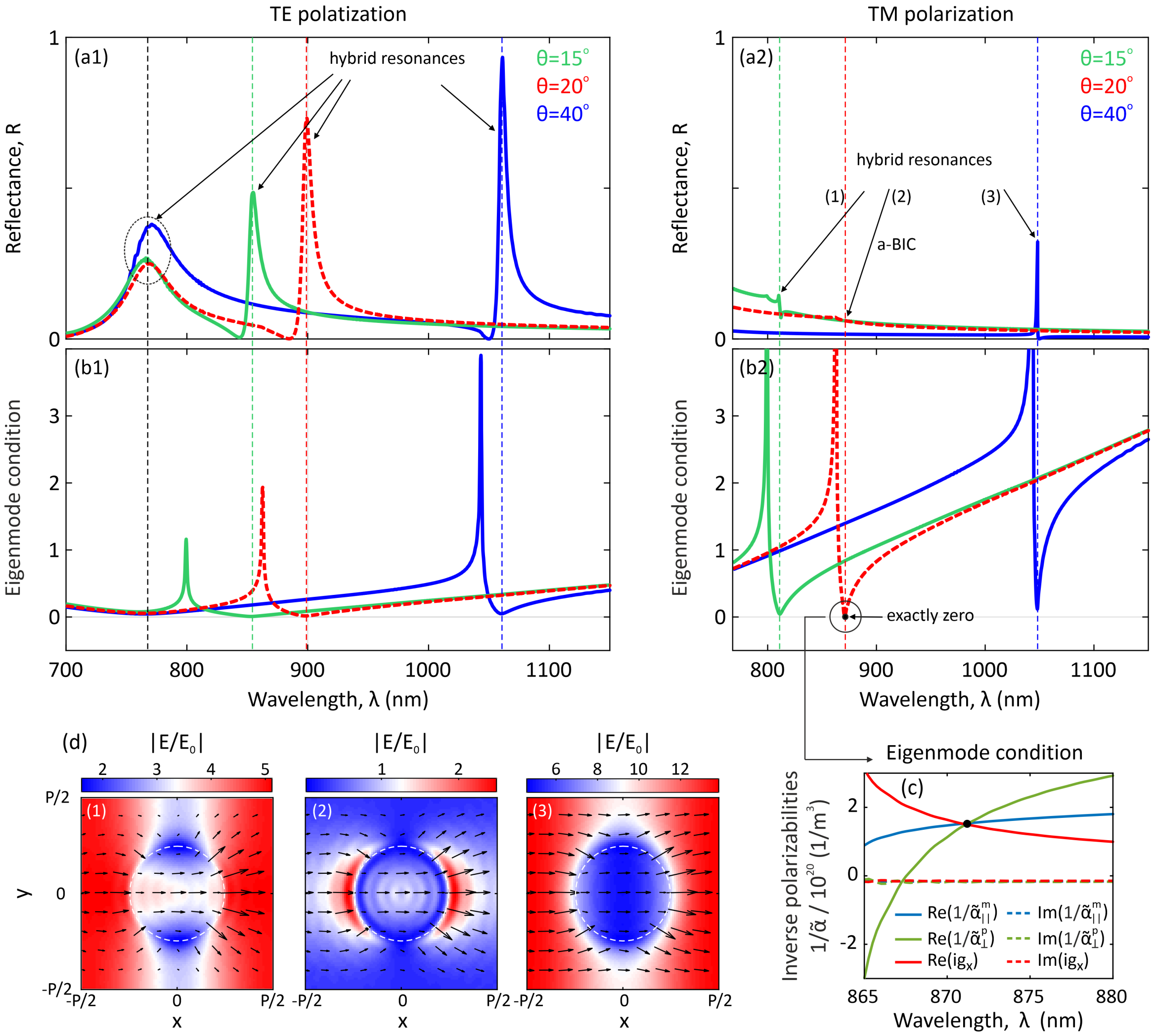

Figure S6 (a) Reflectance and (b) eigenmode conditions at three different angles of incidence for (1) TE- and (2) TM-polarised excitation. The eigenmode conditions are $(1/\alpha_{\|}^{\mathrm{p}} - S_y)(1/\alpha_{\perp}^{\mathrm{m}} - S_z) + g_x^2 = 0$ for TE, and $(1/\alpha_{\|}^{\mathrm{m}} - S_y)(1/\alpha_{\perp}^{\mathrm{p}} - S_z) + g_x^2 = 0$ for TM case. The reflectance resonances correspond to the minima of the eigenmode conditions but have non-zero bandwidth since the conditions are not satisfied (i.e., $\neq 0$). When they are satisfied, e.g., for $\theta = 20^\circ$ in the panel (b2), the resonant state becomes a nonradiative eigenstate [also known as an accidental BIC (aBIC)] of the system with zero bandwidth (and infinite Q-factor). (c) Visualization of the condition for aBIC, i.e., $1/\tilde{\alpha}_{||}^{\mathrm{m}} = 1/\tilde{\alpha}_{\perp}^{\mathrm{p}} = ig_x$, where $1/\tilde{\alpha}_{||}^{\mathrm{m}} = (1/\alpha_{||}^{\mathrm{m}} - S_y)\sin\theta$ and $1/\tilde{\alpha}_{\perp}^{\mathrm{p}} = (1/\alpha_{\perp}^{\mathrm{p}} - S_z)/\sin\theta$ [1, 2]. Under these conditions, the in-plane MD and out-of-plane ED radiation interfere destructively in the far-field region, completely cancelling each other out. (d) The field distributions at the wavelengths indicated by the arrows in panel (a2). Accidental BIC cannot be excited by an external field of a plane wave as one can see from the field distributions in (d), i.e., the electric field is much smaller at the condition of the aBIC [see (2) in (d)] than quasi-aBIC (excited) cases [see (1) and (3) in (d)].

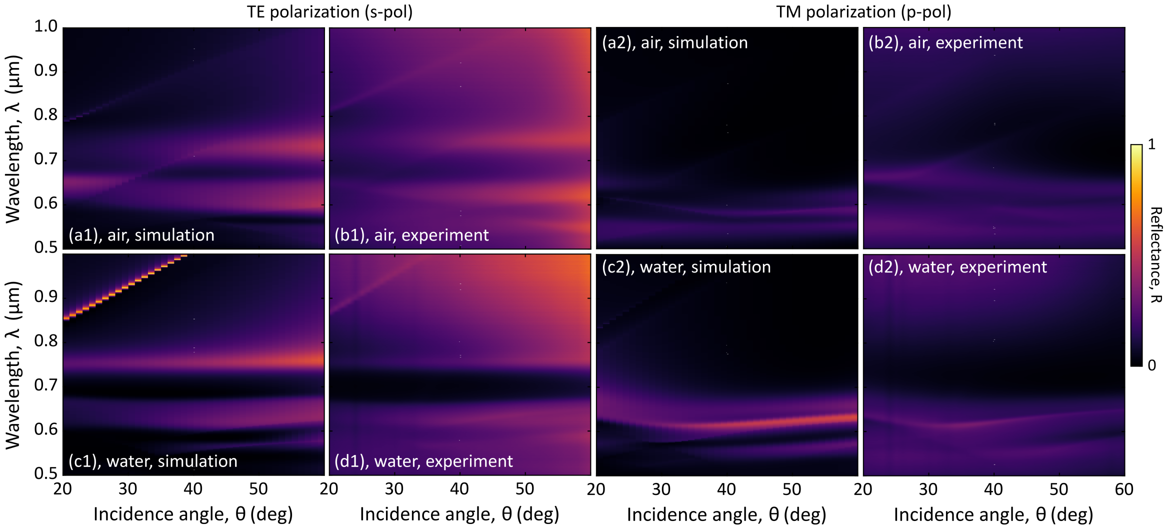

Figure S7 Comparison of (a,c) simulated and (b,d) experimental reflectance dispersion of the metasurface on a glass substrate in the case of (top) air and (bottom) water superstrate for (1) TE and (2) TM polarisations.

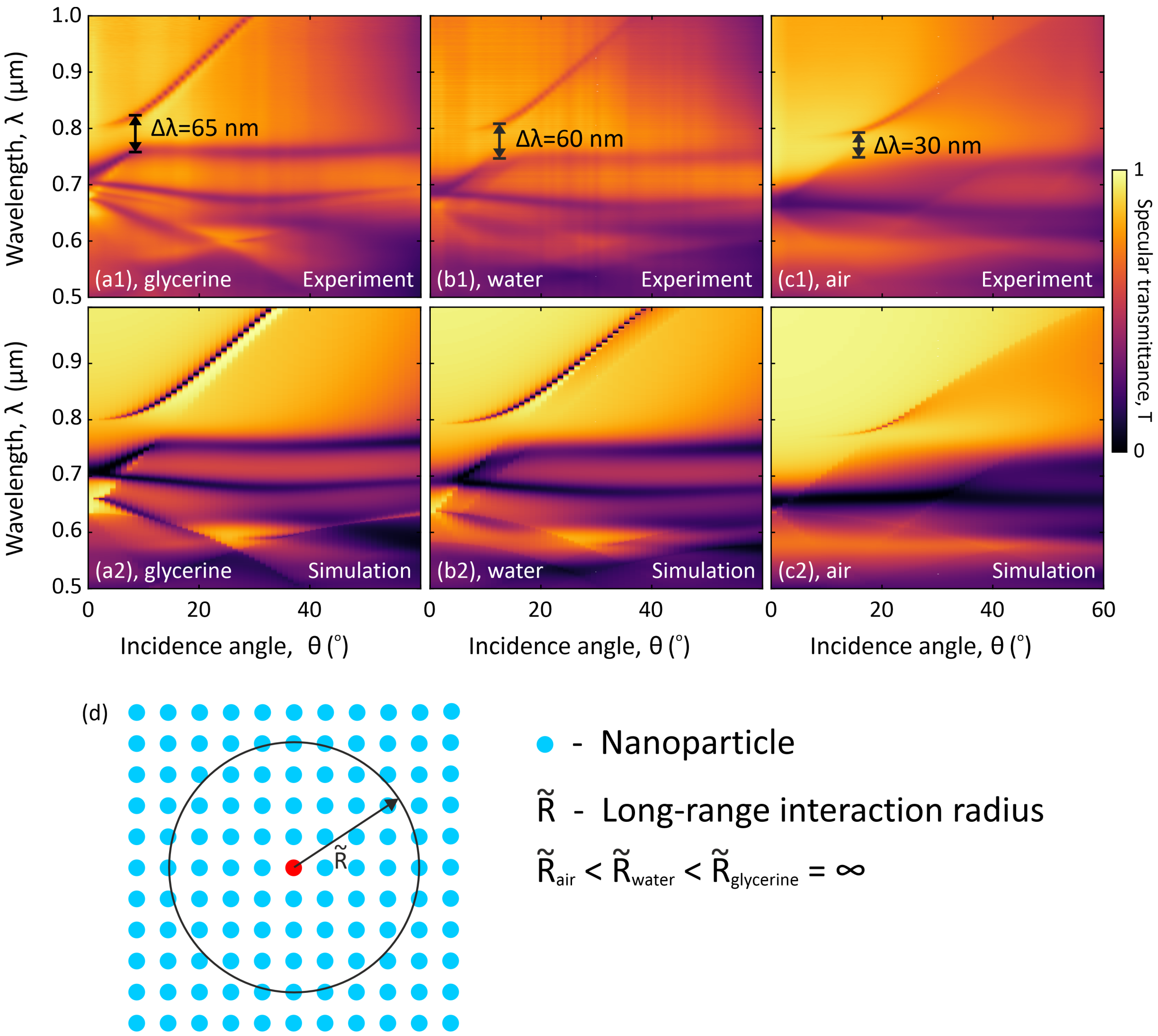

Figure S8 Measured (top row) and simulated (bottom row) specular transmittance of the metasurface on a glass substrate and with (a) glycerine, (b) water, and (c) air superstrate for the TE-polarised illumination. $\Delta\lambda$ is the anticrossing gap, which shrinks with the increase of the substrate-to-superstrate index contrast $\Delta n = n_{\mathrm{sub}} - n_{\mathrm{sup}}$. This can be explained considering the lattice sums S associated with the electromagnetic interaction between metasurface dipoles of the same order, which can be represented as a sum of near-, middle-, and far-field contributions. The far-field part, in contrast to the other two, diverges at the Rayleigh anomaly wavelength (λ^{RA}) in the case of homogeneous surroundings ($\Delta n = 0$). For inhomogeneous surroundings ($\Delta n \neq 0$), the far-field part of S becomes finite at λ^{RA}, as has been shown in Ref. [3]. This is due to the far-field interaction between the nanoparticles being suppressed as a consequence of reflection at the substrate-superstrate interface. (d) Schematic of an array of nanoparticles placed on a glass substrate, indicating the long-range interaction radius $\tilde{R}$ between the nanoparticles, which is inversely proportional to Δn [3]. For the glycerine superstrate (homogeneous surrounding), $\tilde{R} = \infty$ and, therefore, each particle interacts with all particles of the metasurface. For the cases of water and air superstrate, $\tilde{R}$ is finite, and each particle interacts with a finite number of particles around it. The splitting gap $\Delta\lambda$ becomes smaller for a higher index contrast Δn since a limited number of particles participate in the interaction, resulting in a weaker coupling than in homogeneous surroundings.

References

[1] Allayarov, I., Tuz, V. R., Calà Lesina, A. & Evlyukhin, A. B. Analytical model of metasurfaces comprising meta-atoms with anisotropic polarizabilities and for arbitrary incident angles. *Physical Review B* **111**, 155438 (2025).

[2] Abujetas, D. R., Olmos-Trigo, J. & Sánchez-Gil, J. A. Tailoring accidental double bound states in the continuum in all-dielectric metasurfaces. *Advanced Optical Materials* **10**, 2200301 (2022).

[3] Allayarov, I. *et al.* Dynamic nonlocal dielectric metasurfaces: Tuning collective lattice resonances via substrate–superstrate permittivity contrast. *Advanced Photonics Research* **5**, 2300268 (2024).
